# A Case of Upper Tract Urothelial Carcinoma With Neuroendocrine Differentiation Successfully Treated With Enfortumab Vedotin and Pembrolizumab

**DOI:** 10.1002/iju5.70149

**Published:** 2026-02-19

**Authors:** Kosei Taniguchi, Mamoru Hashimoto, Takahito Nakayama, Saizo Fujimoto, Shingo Toyoda, Takashi Kikuchi, Marco Antonio De Velasco, Osamu Maenishi, Takafumi Minami, Kazutoshi Fujita

**Affiliations:** ^1^ Department of Urology Kindai University Faculty of Medicine Osaka Japan; ^2^ Department of Pathology Kindai University Faculty of Medicine Osaka Japan

**Keywords:** Enfortumab vedotin, Nectin‐4, neuroendocrine differentiation, pembrolizumab, upper tract urothelial carcinoma

## Abstract

**Introduction:**

Upper tract urothelial carcinoma with neuroendocrine differentiation (UC‐NE) is extremely rare and generally associated with aggressive behavior and poor prognosis. Optimal treatment strategies remain unclear, particularly regarding the role of nectin‐4–targeted therapy.

**Case Presentation:**

A 61‐year‐old man was diagnosed with UC‐NE of the renal pelvis. Laparoscopic nephroureterectomy revealed invasive UC‐NE with lymphatic invasion (pT1, G2) and carcinoma in situ of the ureter (pTis, G1). Immunohistochemistry showed strong nectin‐4 expression in the urothelial component but only weak to moderate expression in the neuroendocrine component. Ten months after surgery, para‐aortic and bilateral pelvic lymph node recurrence developed. Treatment with enfortumab vedotin (EV) plus pembrolizumab achieved a complete response after 3 cycles, and remission was maintained with continued therapy.

**Conclusion:**

This case suggests that EV plus pembrolizumab may be effective for UC‐NE and highlights the importance of evaluating nectin‐4 and the tumor immune microenvironment when considering treatment strategies for this rare subtype.

## Summary

- Upper tract urothelial carcinoma with neuroendocrine differentiation is exceedingly rare and associated with unclear treatment strategies.
- Although the neuroendocrine component demonstrated weak to moderate nectin‐4 expression, enfortumab vedotin plus pembrolizumab induced a complete response.
- This case indicates that enfortumab vedotin plus pembrolizumab may be effective for urothelial carcinoma with neuroendocrine differentiation.

**Abbreviations**
CT: computed tomography
EV: enfortumab vedotin
IHC: immunohistochemistry
UC: urothelial carcinoma
UC‐NE: urothelial carcinoma with neuroendocrine differentiation
UTUC: upper tract urothelial carcinoma

## 1 Introduction

Urothelial carcinoma (UC) is classified into upper tract urothelial carcinoma (UTUC) and lower tract UC. UTUC is relatively uncommon accounting for only 5%–10% of all UC cases and is associated with a poorer prognosis [1, 2]. Within this subset, UC with neuroendocrine differentiation (UC‐NE) is exceedingly rare. Such tumors are frequently diagnosed at advanced stages and are linked to unfavorable clinical outcomes [3].

For neuroendocrine malignancies, cisplatin plus etoposide has been regarded as the standard regimen. However, survival in patients with neuroendocrine bladder cancer remains extremely poor even with this approach [4]. Notably, the efficacy of enfortumab vedotin (EV), an antibody–drug conjugate targeting nectin‐4, largely depends on the level of nectin‐4 expression. Previous studies have demonstrated that nectin‐4 expression is usually minimal or absent in bladder cancer with neuroendocrine differentiation [5, 6], raising concerns about the therapeutic applicability of EV in this histologic subtype.

Recently, the combination of EV and pembrolizumab has emerged as a promising first‐line therapy for advanced UC [7]. However, its effectiveness in UTUC harboring neuroendocrine features has not yet been reported. Here, we present a rare case of UC‐NE arising in the upper urinary tract, which recurred shortly after surgery but subsequently achieved a durable remission with EV plus pembrolizumab despite limited nectin‐4 expression.

## 2 Case Presentation

A 61‐year‐old man was referred to our department due to the microscopic hematuria during routine health screening. Urinary cytology revealed class IV suspicious cells, but cystoscopy did not detect any bladder tumors. Laboratory findings were unremarkable except for microscopic hematuria (10–19 RBC/HPF). The patient had no history of chronic kidney disease, diabetes mellitus, or other renal dysfunction.

**FIGURE 1:**
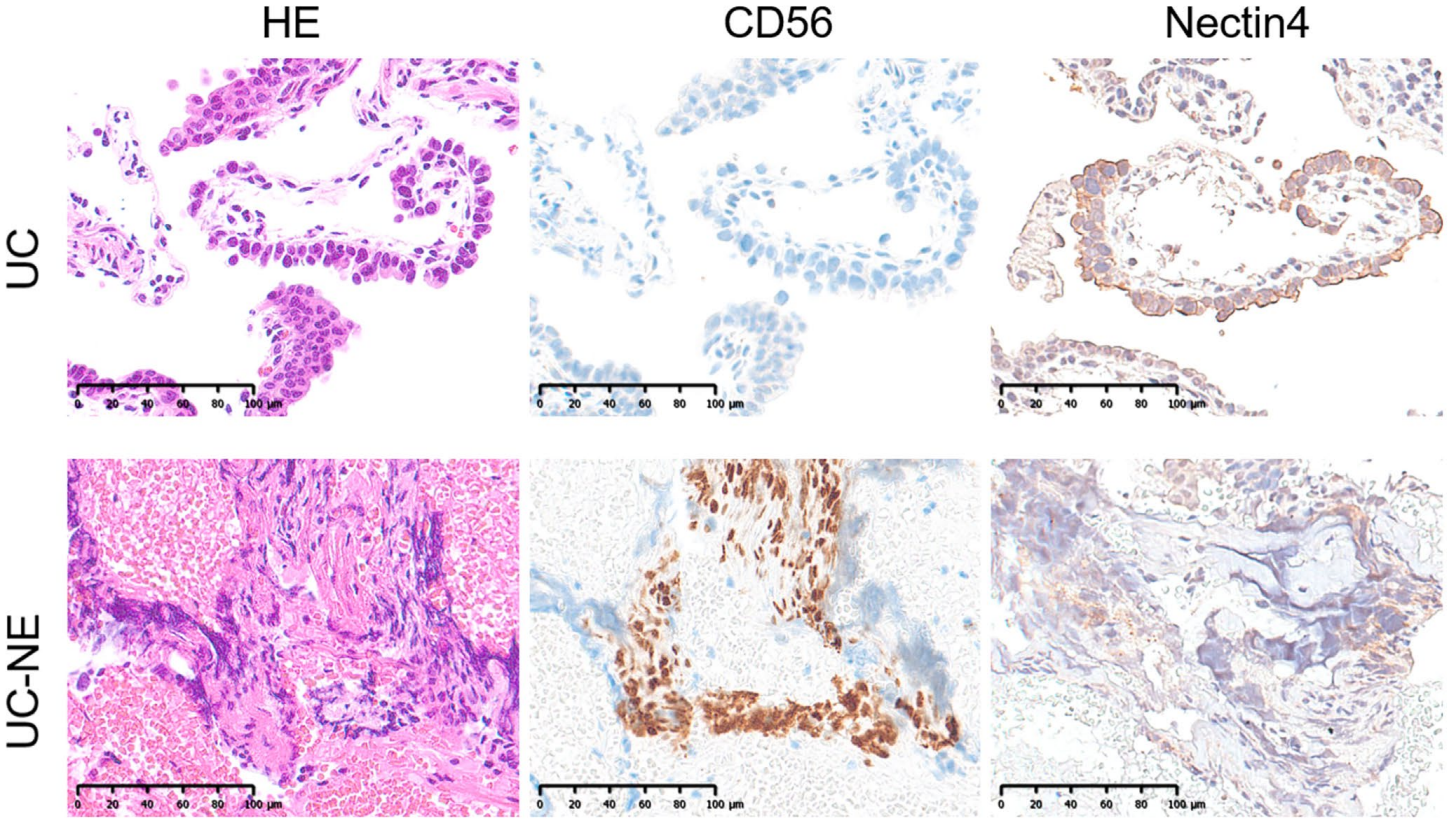
Immunohistochemical staining of the tumor. Representative photomicrographs demonstrate distinct expression profiles: The urothelial carcinoma (UC) component was negative for CD56 and strongly positive for nectin‐4, whereas the UC with neuroendocrine differentiation (UC‐NE) component showed strong CD56 positivity and weak to moderate nectin‐4 staining.

Contrast‐enhanced CT showed no apparent lesions, and retrograde pyelography revealed no filling defects. Ureteroscopy, however, demonstrated a papillary tumor localized in the left renal pelvis. Urinary cytology was suspicious in the left renal pelvis/ureter and negative in the right. Ureteroscopic biopsy confirmed the diagnosis of UC‐NE. The tumor predominantly showed typical UC morphology, but focal areas of small cells with nuclear atypia were present. Immunohistochemistry (IHC) demonstrated positivity for CD56 and INSM1, confirming neuroendocrine differentiation. The percentage of neuroendocrine features per cancer cells populations was 16% based on Ai calculation. No distant metastases were identified, and the clinical stage was cT2N0M0. The patient underwent laparoscopic left nephroureterectomy. Invasive UC‐NE of the renal pelvis with lymphatic invasion (pT1, G2) and carcinoma in situ in the left ureter (pTis, G1), without vascular invasion. IHC revealed that the UC area was negative for CD56 but showed strong positivity for nectin‐4. In contrast, the UC‐NE area exhibited strong CD56 positivity and weak to moderate nectin‐4 staining (Figure 1). Nectin‐4 IHC was performed using an anti‐Nectin‐4 monoclonal antibody (dilution 1:1600, clone EPR15613‐68, Abcam, Cambridge, UK). Template‐based lymph node dissection was omitted because the disease was clinically node‐negative, and limited para‐aortic lymph node sampling revealed no malignancy. The patient was placed on surveillance without adjuvant therapy.

**FIGURE 2:**
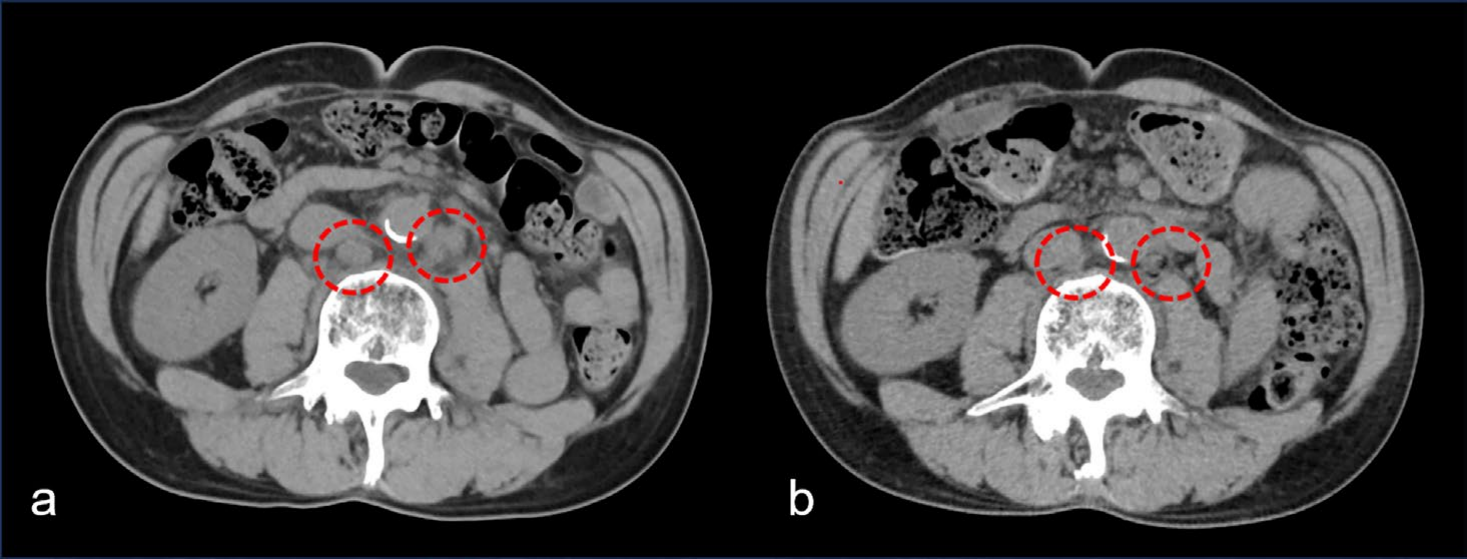
CT images showing recurrent lymphadenopathy and response to systemic therapy. Ten months after surgery, CT scans revealed enlarged para‐aortic and bilateral pelvic lymph nodes (a). Following three cycles of enfortumab vedotin plus pembrolizumab, the metastatic lymph nodes showed marked reduction in size consistent with a complete response to systemic therapy (b).

Ten months after surgery, follow‐up CT revealed multiple enlarged lymph nodes in the para‐aortic and bilateral pelvic regions, consistent with recurrence (Figure 2a). At the time of recurrence, neuroendocrine tumor‐related serum markers were evaluated, revealing a neuron‐specific enolase (NSE) level of 8.2 ng/mL and a pro–gastrin‐releasing peptide (proGRP) level of 48 pg/mL, neither of which was elevated. No visceral or bone metastases were observed. Systemic therapy with EV plus pembrolizumab was initiated. After three cycles, CT showed a significant reduction in metastatic lymph node size, consistent with a complete response (Figure 2b). After 13 cycles (~12 months), remission was maintained. Adverse events included Grade 2 alopecia and Grade 2 ocular dryness, both manageable with supportive care. No severe adverse events occurred.

## 3 Discussion

This case represents a rare case of UC‐NE of the renal pelvis with early nodal recurrence that responded remarkably to EV plus pembrolizumab. The efficacy of EV depends on expression of its target antigen, nectin‐4 [5]. While nectin‐4 expression is generally low in bladder cancer patients with UC‐NE [6], it remains unclear whether patients with UTUC with UC‐NE also exhibit similarly limited nectin‐4 expression. Nectin‐4 is predominantly expressed in luminal subtypes of UC. Previous studies have reported that UTUC patients tend to exhibit luminal rather than basal subtype profiles [8, 9]. However, there are no reports of nectin‐4 expression in UTUC patients with NE differentiation. In our analysis, weak to moderate nectin‐4 staining was observed in the UC‐NE tissue, suggesting that the patient may have been responsive to EV in combination with pembrolizumab therapy.

Typical UC often exhibits an “inflamed” phenotype characterized by abundant intratumoral infiltration of CD8^+^ T cells [10]. In contrast, UC‐NE more frequently displays an “immune‐excluded” pattern, which suggests a potentially limited efficacy of immunotherapy [11]. However, independently of PD‐L1 status, the tumor immune microenvironment plays a critical role in determining treatment responses [10, 11]. Several studies have shown that platinum‐based chemotherapy combined with an immune checkpoint inhibitor is effective in NE tumors, inducing the expansion of CD8^+^ T‐cell clones and correlating with improved survival [12, 13]. Furthermore, inhibition of nectin‐4 signaling not only triggers cytotoxic effects against tumor cells but also activates natural killer cells, enhancing antitumor immunity [14]. A previous study further revealed that T cells are capable of eliminating both antigen‐positive and neighboring antigen‐negative tumor cells through a mechanism known as bystander killing, which helps the clearance of heterogeneous tumors [15]. Based on these findings, it is plausible that an immune response was initiated in the UC regions following EV plus pembrolizumab therapy, which subsequently extended to adjacent UC‐NE regions, contributing to overall tumor control.

Several limitations should be acknowledged. First, we were unable to perform immunohistochemical evaluation of nectin‐4 in the recurrent lymph node lesions, leaving uncertainty about the antigen status in the metastatic sites. Second, it remains possible that the recurrent tumors contained predominantly conventional UC rather than neuroendocrine components, which could have contributed to the favorable response to EV plus pembrolizumab.

In summary, this case underscores the importance of evaluating nectin‐4 expression when considering therapeutic strategies for UC‐NE. Further study is warranted to validate the efficacy of EV plus pembrolizumab therapy in UTUC patients with UC‐NE.

## Conflicts of Interest

The authors declare no conflicts of interest.

## Data Availability

Data sharing not applicable to this article as no datasets were generated or analysed during the current study.
